# A Case of Death Caused by Tracheal Tube Aspiration

**DOI:** 10.5812/aapm.13152

**Published:** 2014-02-25

**Authors:** Alireza Mirkheshti, Morteza Jabbary Moghadam, Mohamad Shahab Kalantar, Dariush Abtahi, Elham Memary

**Affiliations:** 1 Department of Anesthesiology, Shahid Beheshti University of Medical Sciences, Tehran, Iran

**Keywords:** Airway Management, Endotracheal Intubation Complications, Endotracheal Intubation Mortality

## Abstract

**Introduction:**

Airway management, especially outside the operating room, needs meticulous observation in order to avoid certain risks, such as; endotracheal tube (ETT) disloca­tion, esophageal intubation, and unwanted extubation. ETT or tracheostomy dislocation is responsible for one-half of death or brain damage cases in the ICU. Despite appropri­ate fixation of an ETT, the previously mentioned compli­cations can still occur . A broken ETT and consequent airway obstruction may lead to lethal complications.

**Case Presentation:**

We report a case of death caused by tracheal tube aspiration, where it was located distal to the vocal cords, with a part of it entering the right bronchus and the mediastinum, after tearing the right bronchus.

**Discussion:**

The vigilance and experience of medical personnel in the ICU, appropriate IV sedation, and using a bite block are the best ways to prevent mortality caused by aspiration of an ETT in all intubated patients.

## 1. Introduction

Airway management, especially outside the operating room, needs meticulous observation in order to avoid certain risks, such as; endotracheal tube (ETT) dislocation, esophageal intubation, and unwanted extubation.

ETT or tracheostomy dislocation is responsible for one-half of death or brain damage cases in the ICU. These events may happen during; patient’s transportation, discontinuing sedative drugs infusion, abrupt wakening, coughing, or during suctioning of ETT. Despite appropriate fixation of an ETT, the previously mentioned complications can still occur (1). A broken ETT and consequent airway obstruction may lead to lethal complications. 

## 2. Case Presentation

In this report, we present a case of death caused by a broken ETT. Our patient (male) was a 32-year-old, with a known case of Hodgkin's lymphoma of eight years duration, prior to admission to our infectious disease department (Imam Hosein Medical Center, Tehran, Iran) with fever, cachexia, and dyspnea. Antibiotic treatment (imipenem, vancomycin) was started with a diagnosis of pneumonia. After 48 hours from the admission, the patient was transferred to the ICU because of his decreased level of consciousness, and intubation was ordered.

When visited by an anesthesiologist, the patient was confused, cachectic, sweating, and suffering from respiratory distress. Vital signs were recorded as follows: PR = 110 /min, RR = 40/min, axillary temperature = 38°C, and blood pressure = 90/50 mmHg. In lung auscultation, the breathing sounds were muffled in the left superior and middle lobes, and coarse crackles were heard in the lung bases.

Arterial blood gas analysis results were: pH = 7.18, HCO3 = 15 mmol/L, PCO2 = 23 mmHg, PO2 = 48 mmHg, BE = -8 mmol/L O_2_, and saturation = 85%.

Considering the patient’s condition, cardiorespiratory monitoring and pulse oximetry were established. 

After suctioning the oral secretions, an intravenous injection of 150 µg fentanyl and 80 mg lidocaine 2%, plus four puffs of lidocaine 10%, were administered to anesthetize the glossopharyngeal nerve and a direct laryngoscopy was carried out. Then, the patient was intubated with a high volume low-pressure tracheal tube No 8, with an inflated cuff. After the correct position of tube was confirmed, the cuff was filled with 5cc air. 

Mechanical ventilation was established in the synchronized intermittent mandatory ventilation (SIMV) mode with pressure support parameters as follows: RR = 12/min, TV = 450 cc, PEEP = 3 cm H_2_O, pressure support = 15 cm H_2_O, flow trigger = 2 L/min, inspiratory time = 1.5 sec, inspiratory flow rate = 50 L/min, and FIO_2 _= 100%. 

Hemodynamic parameters after intubation were s follows: BP = 100/60, HR = 120/min, and SPO_2_ = 90-92%.

We ordered an intravenous sedation with 1-2 mg midazolam and 50-100 mg fentanyl to be started if the patient's BP fell below 90 mmHg.

After one hour of intubation, the ventilator began to alarm, and the ICU nurse informed us about the disappearance of a part of the tracheal tube.

An anesthesiologist was dispatched immediately to the ICU and determined the disappearance of a part of tracheal tube from around number 22, with bite marks in the remaining portion. The patient was cyanotic and his hemodynamic parameters were unstable. Pulse oximetry identified the O_2_ saturation to be 60%. It was impossible to open the patient’s mouth and although a jaw thrust maneuver was performed, ventilation with a mask was unsuccessful. Direct laryngoscopy did not show any traces of the aspirated part of the tracheal tube, and two consecutive attempts with Magill forceps to extract the tube were unsuccessful. The patient entered cardiorespiratory arrest; hence, reintubation of the patient was performed with tracheal tube number 7. Cardiopulmonary resuscitation was performed for 45 minutes with no success and finally, the patient expired.

A postmortem chest X-ray was requested to evaluate the position of the aspirated piece of tracheal tube. X-ray images showed that it was located distal to the vocal cords, with a part of it entering the right bronchus and the mediastinum, after tearing the right bronchus. The chest X-ray was not of acceptable quality, thus it was impossible to localize the exact location of the ETT. However, no further chest X-ray was performed due to a lack of consent from the patient’s relatives. A postmortem autopsy was performed and it confirmed that the ETT was located distal to the vocal cords, with a part of it entering the right bronchus and the mediastinum after tearing the right bronchus. 

## 3. Discussion

This is the first case report of mortality and rupture of the bronchus after aspiration of an ETT. The aspirated ETT entered the right bronchus and mediastinum after tearing the right bronchus. This event caused airway obstruction and an inability to ventilate.

In reviewing the reports of aspirated ETT, we found only a few case reports. In one report, a 10.5 mm ETT in a 40-year-old patient with a stab wound in the chest area was aspirated (2). The authors managed to reintubate the patient using a 7 mm ETT and the patient was saved from certain death. The chest X-ray images demonstrated that the second tube had entered the lumen of the first tube. In another case, a five-year-old patient who bit the ETT after recovering from anesthesia, had aspirated the whole ETT, which was severed from its attachment to the metal connector (3). In this case, the aspirated part was successfully extracted by using Magill forceps. There was also another report of a 62-year-old woman suffering from papillary carcinoma of the thyroid, who aspirated the ETT without showing any signs of respiratory distress. After performing a chest X-ray, the aspirated tube was found and successfully extracted by performing a tracheostomy in the operating room under local anesthesia (4). 

**Figure 1.**
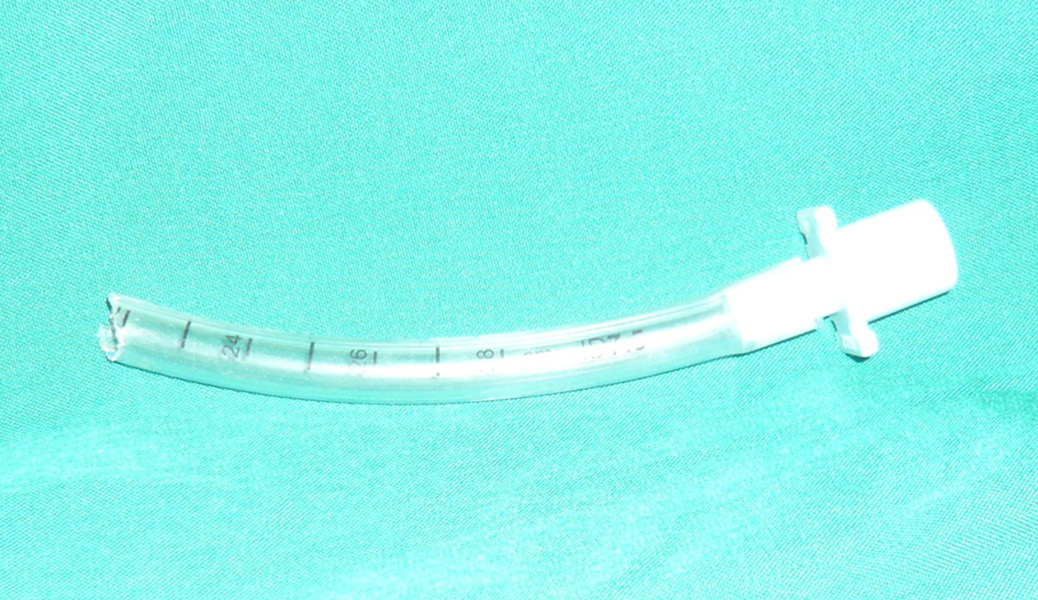
Broken Endotracheal Tube Showing the Bite Site

**Figure 2.**
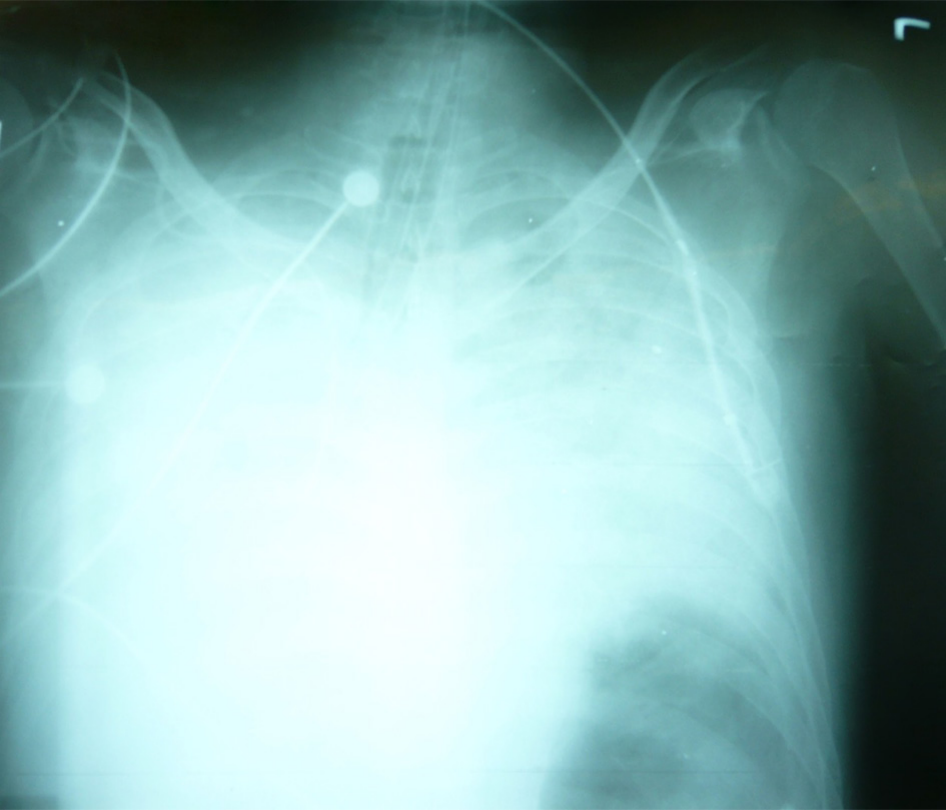
Chest X-Ray of Patients After Second Intubation (Postmortem)

In our case, the ETT was cut at number 22, indicating aspiration of 22 cm of the tracheal tube (Figure 1). As we could not see the tracheal tube from the vocal cord level through the 10-15 cm distance from the vocal cords to the carina, it can be hypothesized that the end part of the aspirated tube had passed the carina and entered the right bronchus or penetrated the tracheal and entered the mediastinum (Figure 2). 

In most cases, the diagnosis is confirmed by chest radiography (5). Computed tomography of the chest may also be valuable in identifying small aspirated objects, or when an associated chest disease is suspected. Bronchoscopy is frequently used both for diagnostic and therapeutic purposes (6, 7). The availability of both rigid and flexible bronchoscopy should be emphasized, since larger aspirates may not be retrievable with a flexible bronchoscope (7). In our case, there was no opportunity to perform a bronchoscopy, due to the rapid deterioration of the patient’s cardiorespiratory condition. Hence, we attempted reintubation after an unsuccessful laryngoscopy, as a last resort. This second attempt might have caused the first aspirated tube to move, and then enter the mediastinum, before tearing the bronchus. Therefore, we can conclude that whenever possible, the aspirated ETT should be extracted before any attempt to reintubate the patient in order to avoid further complications of the patient’s respiratory condition. 

In one case report, authors were successful in reintubation, without extracting the aspirated tube, with a second tube, as it was a smaller size than the primary tube (2). Therefore, if the patient has entered respiratory arrest and all possible attempts to find and extract the aspirated tube have failed, reintubation could be attempted with a tube of smaller diameter than the aspirated one, in a worst case scenario. Nevertheless, the success of this method has been recorded in only one case report and the success rate remains unclear. In conclusion, the vigilance and experience of medical personnel in the ICU, appropriate IV sedation, and using a bite block are the best ways to prevent mortality caused by aspiration of an ETT in all intubated patients.
